# Analysis of HMF and furfural in hydrolyzed lignocellulosic biomass by HPLC-DAD-based method using FDCA as internal standard

**DOI:** 10.1016/j.mex.2022.101774

**Published:** 2022-06-25

**Authors:** Cristian Acker Godoy, Patrícia Valderrama, Andreia Cristina Furtado, Marcela Boroski

**Affiliations:** a Federal University of Latin American Integration (UNILA), Av. Tancredo Neves, 6731, Foz do Iguaçu, PR, 85867-970, Brazil; b Federal Technological University of Paraná (UTFPR), Via Rosalina Maria dos Santos, 1233, Campo Mourão, PR, 87301-899, Brazil

**Keywords:** Food analysis, Lignocellulosic biomass hydrolysis, Biomass residue analysis, 2,5-Furandicarboxylic acid, 5-Hydroxymethylfurfural

## Abstract

HMF (hydroxymethylfurfural), a compound that occurs naturally in food, is derived from the dehydration of monosaccharides (glucose and fructose) in products subjected to high-temperature treatments or to aging processes. HMF can be obtained by acid-catalyzed dehydration of lignocellulosic biomasses such as sugarcane bagasse and other agricultural residues. In this work, analytical quantification of HMF and furfural (the main co-product) was performed using high-performance liquid chromatography coupled with diode array detection (HPLC-DAD). The official method employs isocratic elution with a mobile phase composed of water and acetonitrile at a ratio of 80:20 (v/v). The analytical method proposed here was developed using 2,5-furandicarboxylic acid (FDCA) as internal standard, for the first time, with 0.01 mol L^−1^ trisodium citrate and ultrapure water as the mobile phase, adjusted to pH 2.5. The acidity of the mobile phase was required to avoid FDCA deprotonation. Good peak resolution and selectivity were obtained, without differences in the retention times of the analytes present in the standard solutions used to obtain the analytical curve and in the aqueous and organic phases from the synthesis of HMF using lignocellulosic biomass hydrolysis. The method complies with the current recommendations of AOAC regarding validation parameters.

• *The proposed HPLC method improves peak selectivity and resolution.*
• *The method is suitable for acid sample media, such as aqueous and organic hydrolysis phases.*
• *2,5-Furandicarboxylic acid (FDCA) was used as internal standard.*

## Graphical abstract

**Figure:**
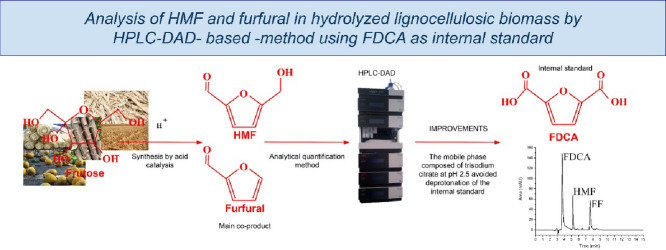

Specifications table

**Table:** 

Subject Area:	Chemistry
More specific subject area:	*Food and biomass analysis*
Method name:	*HPLC-DAD-based method for HMF and furfural using FDCA as internal standard*
Name and reference of original method:	J.K. de Andrade, E. Komatsu, H. Perreault, Y.R. Torres, M.R. da Rosa, M.L. Felsner, In house validation from direct determination of 5-hydroxymethyl-2-furfural (HMF) in Brazilian corn and cane syrups samples by HPLC-UV. Food Chem. 190 (2016) 481–486. https://doi.org/10.1016/j.foodchem.2015.05.131
Resource availability:	Reagents: - Type I ultrapure water with resistivity of 18.2 MΩ.cm (PURELAB Option Q system) - Trisodium citrate buffer solution (Sigma-Aldrich, MW 294.10 g mol^−1^, purity ≥99.0%) - Glacial acetic acid (Dinâmica, UV/HPLC grade, purity of 99.5%) - HMF (Sigma-Aldrich, MW 126.11 g mol^−1^, purity ≥99.0%) - Furfural (Sigma-Aldrich, MW 96.08 g mol^−1^, purity of 99.0%) - FDCA (Sigma-Aldrich, MW 156.09 g mol^−1^, purity of 97.0%) Materials: - pH meter (mPA210, MS Tecnopon)

## Method details

### Solution preparations

**Citrate buffer solution**: Trisodium citrate buffer solution at 0.01 mol L^−1^ was prepared by solubilizing 11.7 g in 4000 mL of ultrapure water. The pH was adjusted to 2.5 with approximately 600 mL of glacial acetic acid, using a pH meter.

**HMF stock solution**: HMF at 1.25 mmol L^−1^ was prepared by solubilizing 0.0158 g of the analytical standard in 100 mL of ultrapure water.

**Furfural (FF) stock solution**: FF at 2.50 × 10^−1^ mmol L^−1^ was prepared by dilution of 16.6 μL of the analytical standard in 100 mL of ultrapure water.

**2,5-Furandicarboxylic acid (FDCA) stock solution**: FDCA at 2.00 × 10^−1^ mmol L^−1^ was obtained by diluting 16.70 mL of a solution at 1.20 mmol L^−1^ (0.0188 g of the analytical standard in 100 mL of ultrapure water) in 100 mL of ultrapure water.

### HPLC parameters

The separation and quantification of the analytes of interest were carried out using a high-performance liquid chromatography (HPLC) system (Dionex UltiMate 3000 series, Thermo Fisher Scientific), coupled to a diode array detector (DAD 3000) and equipped with a quaternary pump (LGP-3400SD) and an autosampler. The analysis was performed in isocratic elution mode, with a mobile phase composed of trisodium citrate buffer solution (pH 2.5), pumped through the column at a constant flow rate of 1 mL min^−1^. The chromatographic separation employed an ACE 5 C18 column (batch no. V13-7473) (250 mm × 4.6 mm; particle size of 5 μm; particle porosity of 110 Å), kept at 30 °C. The injection volume was 20 μL. The diode array detector (DAD) recorded the spectra in the range from 200 to 400 nm, with detection of the analytes at specific wavelengths of 263, 277, and 285 nm for FDCA, FF, and HMF, respectively.

**Fig. 1:**
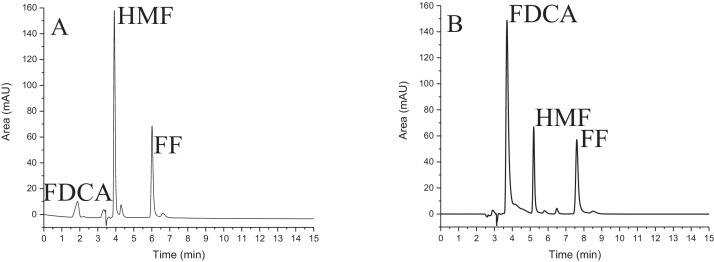
Isocratic elution using the mobile phase composed of water and acetonitrile (80:20, v/v), at neutral pH (A), and using the mobile phase composed of 0.01 mol L^−1^ trisodium citrate solution at pH 2.5 (B). The solutions contained FDCA at a concentration of 2.00 × 10^−2^ mmol L^−1^, and HMF and FF standards at concentrations of 1.00 × 10^−2^ mmol L^−1^. The retention times of the compounds were 3.7 min for FDCA, 5.2 min for HMF, and 7.6 min for FF.

*Consideration regarding mobile phase composition:* Chromatographic methods for the analysis of synthesized HMF and FF in samples from acid-catalyzed dehydration of lignocellulosic biomass were previously investigated in our laboratory [1] using external standardization [2], employing a mobile phase consisting of water:acetonitrile (80:20, v/v). In the present work, a study was conducted to evaluate the potential application of 2,5-furandicarboxylic acid (FDCA) as internal standard, with the aim of mitigating the oscillations of the HPLC-DAD equipment. The chromatogram shown in Fig. 1A displays the profiles of FDCA, HMF, and FF, obtained using water and acetonitrile (at neutral pH) as the mobile phase. Under this condition (neutral pH), when FDCA was added as internal standard to the relatively acidic synthesis product of lignocellulosic biomass hydrolysis, the FDCA peak presented lack of symmetry. Fig. 1B shows the chromatogram for elution of the analytes (FDCA, HMF, and FF) when 0.01 mol L^−1^ trisodium citrate solution (at pH 2.5) was used as the mobile phase. Due to the chemical structure of FDCA, variations in pH can cause changes related to deprotonation, leading to alteration of the interaction of the compound with the stationary phase, as well as affecting the elution time. FDCA is a carboxylic acid that has hydroxyls attached to each carbonyl. Considering the process of deprotonation, FDCA can stabilize the negative charge by means of resonance and, as such, it is easily deprotonated; this can be seen from its pKa value (2.28).

## Method validation

### Linear range

The analytical curves were constructed using six points, in triplicate. Two curves were prepared for each of the analytes, with the aim of covering the working range. The HMF and FF curves were prepared using identical concentrations at low level (5.00 × 10^−4^ to 3.00 × 10^−2^ mmol L^−1^) and high level (3.00 × 10^−2^ to 2.1 × 10^−1^ mmol L^−1^). A volume of 50 mL of each solution was prepared, with the addition of 5.00 mL of FDCA at 2.00 × 10^−1^ mmol L^−1^ and 500 μL of HCl at 0.50 mol L^−1^ to acidify the solution. The final concentration of the internal standard (FDCA) was 2.00 × 10^−2^ mmol L^−1^ in the solutions used for the analytical curves, as well as in the samples investigated. The analytical curves presented linearity at 95, 99, and 99.9% confidence intervals, while the residuals graph showed homoscedasticity, with a random distribution of the residuals. Furthermore, the linearity was evaluated by comparing the residuals of the linear fit and the quadratic adjustment by using an F-test [3,4].

### Limits of detection (LOD) and quantification (LOQ)

The LOD and LOQ values were calculated mathematically using the validation worksheet elaborated by Ribeiro et al. [3], where the estimation was based on the confidence interval of the regression. The analytical signal estimation from the regression equation presents a standard error, in which the product of this error by the appropriate t value of Student's distribution allows to calculate the confidence interval of the analytical curve. This confidence interval has a form of two hyperbolic lines around the curve. The intercept of the upper limit of the confidence interval is known as the y critical and its projection on the lower limit is an estimate of the minimum concentration that can be measured with a proven degree of confidence statistically, i.e., the LOD. In the same way, LOQ was estimated according to the confidence interval of the regression. For this, a concentration (xc) is pointed where the intercept of the analytical curve touches the lower limit. Then, an yh value is defined as the projection of the xc to the upper limit of confidence. Finally, the yh projection to the lower limit is defined as the LOD value [3,4].

### Precision

For the study of repeatability, ten replicates were prepared using HMF concentrations of 4.8 × 10^−2^, 1.2 × 10^−1^, and 1.92 × 10^−1^ mmol L^−1^, and FF concentrations of 3.45 × 10^−3^, 1.53 × 10^−2^, and 2.71 × 10^−2^ mmol L^−1^. For the analysis of intermediate precision, employing the same preparation methodology as in the repeatability analysis, ten replicates were prepared by the same analyst on two separate days, in the same laboratory and using the same equipment.

### Recovery rate (percentage)

Solutions were prepared using HMF synthesis samples fortified with the standards of the analytes investigated. A 1% (v/v) solution of the synthesis sample was used as matrix solution. A solution containing 50 μL of the synthesis sample and 500 μL of FDCA at 2.00 × 10^−1^ mmol L^−1^ was employed. Aliquots of the HMF and FF stock solutions were added to the mixture, in 5 mL volumetric flasks, and the volume was completed with ultrapure water. For the recovery analysis, it was not necessary to add 0.50 mol L^−1^ HCl to the mixture, since the volume of the synthesis sample added was sufficient to adjust the pH to a value similar to that obtained with the addition of HCl. The synthesis sample employed in this analysis (a sample of the aqueous phase obtained from sugarcane bagasse biomass hydrolysis) was chosen in order to cover the most frequent concentration ranges. The sample was fortified with HMF and FF at the concentrations used in the repeatability and intermediate precision analyses.

### Analysis of real samples

A 10 μL aliquot of synthesis sample was added to a volume of 890 μL of ultrapure water and 100 μL of FDCA at 2.00 × 10^−1^ mmol L^−1^, followed by homogenization (vortex) for 10 s. The samples were passed through 0.22 μm polytetrafluoroethylene (PTFE) hydrophilic syringe filters (Analítica) and were then kept at -20 °C, prior to the chromatographic analyses. For each of the three synthesis replicates, three sample replicates in the aqueous phase and three sample replicates in the organic phase were prepared for injection into the HPLC system.

Table 1 presents the validation parameters obtained for the curves that were most frequently used with the samples, with the high-level curve for HMF and the low-level curve for FF. Two curves were prepared so as to fully account for the concentrations obtained in the samples and to enable evaluation of the linear range of the method. The correlation coefficients obtained were higher than 0.997. For all the analytical curves, the graphs of the residuals exhibited randomly distributed errors. The values obtained for the limits of detection and quantification were lower than the analyte concentrations found in the samples. The repeatability values were below 7.3%, in compliance with the AOAC guidelines [5] for analytes at 10 ppm. In addition, the intermediate precision values were consistent with the Horwitz ratio [5], which should be smaller than 1.3 for inter-day precision evaluation. The recovery percentages for samples containing analytes at concentrations ranging from 10 ppm to 100 ppb should be between 80 and 110%. Therefore, the recovery percentages obtained for the samples analyzed in this study were in excellent compliance with the AOAC guidelines [5].

**Table 1:** Validation parameters for the methodology used to quantify HMF and FF.

Analyte	Linear range (mmol L^−1^)	Equation of the line	R^2^	LOD (mmol L^−1^)	LOQ (mmol L^−1^)	Level (mmol L^−1^)	Rep. RSD(%) n = 10	Int. prec. RSD(%) n=10	Rec. RSD(%) n = 3
HMF	3.00 × 10^−2^ 2.10 × 10^−1^	y = (x-0.5562)/ 50.2139	0.9972	0.002371	0.035249	0.048	0.83	2.89	98.76
						0.120	0.84	3.97	98.63
						0.192	3.90	3.34	97.26
FF	5.00 × 10^−4^ 3.00 × 10^−2^	y = (x-0.0011)/ 50.5597	0.9998	0.000855	0.012651	0.0035	0.72	0.41	97.80
						0.0153	1.23	1.63	94.56
						0.0271	4.46	5.87	91.12

R^2^ = coefficient of determination; LOD = limit of detection; LOQ = limit of quantification; RSD = relative standard deviation; Rep. = repeatability; Int. prec. = intermediate precision; Rec. = recovery.

## Declaration of Competing Interest

The authors declare that they have no known competing financial interests or personal relationships that could have appeared to influence the work reported in this paper.

## Data Availability

Data is available in the paper.
